# Analysis of Hydrogen Generation through Thermochemical Gasification of Coconut Shell Using Thermodynamic Equilibrium Model Considering Char and Tar

**DOI:** 10.1155/2014/654946

**Published:** 2014-11-04

**Authors:** Shanmughom Rupesh, Chandrasekharan Muraleedharan, Palatel Arun

**Affiliations:** Department of Mechanical Engineering, National Institute of Technology Calicut, Calicut, Kerala 673601, India

## Abstract

This work investigates the potential of coconut shell for air-steam gasification using thermodynamic equilibrium model. A thermodynamic equilibrium model considering tar and realistic char conversion was developed using MATLAB software to predict the product gas composition. After comparing it with experimental results the prediction capability of the model is enhanced by multiplying equilibrium constants with suitable coefficients. The modified model is used to study the effect of key process parameters like temperature, steam to biomass ratio, and equivalence ratio on product gas yield, composition, and heating value of syngas along with gasification efficiency. For a steam to biomass ratio of unity, the maximum mole fraction of hydrogen in the product gas is found to be 36.14% with a lower heating value of 7.49 MJ/Nm^3^ at a gasification temperature of 1500 K and equivalence ratio of 0.15.

## 1. Introduction

Gasification is a thermochemical process by which low energy density fuels like biomass can be converted into gaseous fuels with the aid of a series of chemical reactions. One of the main components of gaseous fuel obtained from gasification is hydrogen, which can be used in internal combustion engines and fuel cells. Hydrogen is a clean fuel and energy released by it on combustion is higher than any other fuel on mass basis [1]. Thus it can be considered as a suitable solution for problems associated with fossil fuel depletion and global warming, if its availability is ensured from a sustainable source. Being a renewable energy source, biomass can be considered as a potential candidate for hydrogen production by gasification. Hydrogen yield from biomass gasification depends on several factors like moisture content, feed stock composition, type of gasifier, amount of gasifying agent, and so forth. Influence of different gasifying agents on product gas distribution was studied by Gil et al. [2]. From the study it was found that, compared to air, lower heating value and tar yield are high when steam was used as gasifying agent. Critical component of any gasification system is the gasifier where the homogeneous and heterogeneous reactions take place. A comparison between fixed bed and fluidised bed gasifiers was made by Warnecke [3]. It was concluded that in spite of low ash melting point and high dust content in the product gas, fluidised bed gasifiers have higher heat and mass transfer compared to fixed bed, which result in better temperature distribution.

Mathematical models can be used to investigate biomass gasification especially when large scale experimental study seems to be difficult and uneconomical. A detailed review on different gasification models was presented by Puig-Arnavat et al. [4] and D. Baruah and D. C. Baruah [5]. The comparison of different models showed that thermodynamic equilibrium model (TEM) is the simplest and can be used as an effective preliminary tool to study the effect of process parameters and fuels on gasification. Thermodynamic equilibrium model can be implemented through two approaches, namely, stoichiometric and nonstoichiometric [6]. Compared to the former approach the latter one is complex, even though the basic principle of both is one and the same. Thus many researchers formulated stiochiometric equilibrium models for simulating biomass gasification [7–13]. Application of stoichiometric models for air gasification was successfully demonstrated by Zainal et al. [14], and the modification method used to augment the prediction accuracy of similar models was given by Jarungthammachote and Dutta [15]. Modification of thermodynamic equilibrium model, to reduce its deviation from experimental data, is done mainly by considering char conversion, tar formation, and introducing suitable correction factors to equilibrium constants. A Gibb's free energy minimisation model for air gasification was developed by Ghassemi and Shahsavan-Markadeh [16] and the prediction accuracy of the model was improved by incorporating carbon conversion and tar formation from Azzone et al. [17] and Barman et al. [18], respectively. Azzone et al. [17] considered char conversion as a function of equivalence ratio (ER) in air-steam gasification whereas tar was incorporated by Barman et al. [18] in air gasification, as a compound containing carbon, hydrogen, and oxygen. The deviation of stoichiometric model developed by Huang and Ramaswamy [19], from thermodynamic equilibrium, was reduced by multiplying suitable coefficient with equilibrium constants as done by Jarungammachote and Dutta [15] and Loha et al. [11] for air and steam gasifications, respectively. Similar modification was applied by Lim and Lee [20] to air-steam gasification model in which char conversion was expressed as a function of equivalence ratio and temperature. Abuadala et al. [13] included unreacted char as 5% of biomass carbon content and tar as benzene in steam gasification model. A pseudoequilibrium air-steam gasification model with correction factors for equilibrium constants in terms of reactor temperature was developed by Ng et al. [10]. This model considered tar as a compound containing carbon, hydrogen, and oxygen, and char as solid carbon. A three stage quasi-equilibrium model (considering pyrolysis, char-gas reactions and gas phase reactions in each stage) for steam gasification of biomass was developed by Nguyen et al. [21] where empirical relations were used to reduce the deviation from thermodynamic equilibrium. Puig-Arnavat et al. [22] developed a modified equilibrium model for air steam gasification of biomass using Engineering Equation Solver (EES). Deviation of this model from pure equilibrium is minimised by considering pyrolysis, heat loss in pyrolysis, char and tar, and particles leaving the gasifier and setting the amount of CH_4_ produced. Review of the literatures reveals that stoichiometric models formulated for biomass air-steam gasification considering both char and tar are limited. Present work deals with the stoichiometric modeling of air-steam gasification considering tar and char. Coconut shell, a locally available nonedible biomass waste, is the feedstock selected for the study.

## 2. Model Development

In general thermodynamic equilibrium calculations are independent of gasifier design and are suitable for analysing the effect of fuel and process parameters [6]. These models are more appropriate for simulating entrained flow gasifiers in chemical process simulators or for downdraft fixed-bed gasifiers, as long as high temperature and gas residence time are achieved. The objective of present work is to develop a thermodynamic equilibrium model to simulate fluidised bed biomass gasifier.

Biomass gasification being a complex process, its theoretical modeling requires certain assumptions. The assumptions used to formulate the biomass gasification process are as follows.

- Gasifier is considered as a steady state system with uniform temperature and pressure throughout.
- The residence time of the gases in the gasifier is high enough to establish thermodynamic and chemical equilibria.
- All the gases behave ideally.
- Gases except H_2_, CO, CO_2_, CH_4_, and N_2_ are considered dilute.
- N_2_ is considered as inert in the entire process.
- Biomass is considered to be made up of carbon, hydrogen and oxygen.
- Steam is supplied under superheated condition of 1 bar and 300°C.

By considering chemical formula of feedstock as CH_*X*_O_*Z*_, global gasification reaction can be written as

(1) nbCHXOZ+wH2Ol+sH2Og+mO2+3.76mN2 ⟶nH2H2+nCOCO+nCO2CO2+nCH4CH4  +nH2OH2Og+1−αC+ntartar+3.76mN2,

where *X* and *Z* are the number of atoms of hydrogen and oxygen for each atom of carbon per mole of biomass; *w* is the amount of moisture present in dry ash free biomass; *s* and *m* are the amount of steam and oxygen supplied, respectively. On the right hand side, *n*_H_2__, *n*_CO_, *n*_CO_2__, *n*_CH_4__, and *n*_tar_ are the numbers of moles of H_2_, CO, CO_2_, CH_4_, and tar, and *α* is the char conversion factor which can be expressed as a function of equivalence ratio [23], given by

(2) $$\begin{array}{c} \alpha =0.32+0.82\left(1-e^{-\text{ER}/0.229}\right). \end{array}$$

Tar is incorporated in the model as a mixture of benzene, toluene, and naphthalene in 1 : 2.5 : 6.5 proportions by weight [24, 25] and its yield can be obtained as a weight percentage of the total gasification products using the following [20]:

(3) $$\begin{array}{c} {\text{Tar}}_{\text{wt}.\%}=35.98\exp\left(-0.0029T\right), \end{array}$$

where *T* is the temperature.

Total weight of the gasification product is obtained by applying mass balance to the global reaction between the reactants and the products. So mass of tar yield (*m*_tar_) is given by

(4) mtar=Tarwt.%100biomass  feed+SBR∗biomass  feed     s+moisture  in  biomass+air  supplied.

To determine five unknown constituents of the producer gas, five separate equations are required. These equations are developed from mass balance of C, H, and O from (1) and equilibrium constant relations for water gas shift reaction (see (9)) and methane reaction (see (11)):

carbon balance:

(5) nCO+nCO2+nCH4+6nC6H6+7nC7H8 +10nC10H8+1−α−nb=0;

hydrogen balance:

(6) 2nH2+4nCH4+2nH2O+6nC6H6+8nC7H8 +8nC10H8−xnb−2s−2w=0;

oxygen balance:

(7) $$\begin{array}{c} n_{\text{CO}}+2n_{\text{C}{\text{O}}_{2}}+n_{{\text{H}}_{2}\text{O}}-yn_{b}-s-w-2m=0; \end{array}$$

water gas shift reaction:

(8) $$\begin{array}{c} {{\text{CO}+\text{H}}_{2}\text{O}⟶\text{CO}}_{2}+{\text{H}}_{2}. \end{array}$$

Considering equilibrium constant *K*_1_ for water gas shift reaction,

(9) $$\begin{array}{c} K_{1}=\frac{n_{\text{C}{\text{O}}_{2}}n_{{\text{H}}_{2}}}{n_{\text{CO}}n_{{\text{H}}_{2}\text{O}}}. \end{array}$$

Methane Reaction is as follows:

(10) $$\begin{array}{c} {\text{C}+2{\text{H}}_{2}⟶\text{CH}}_{4}. \end{array}$$

Considering equilibrium constant *K*_2_ for methane reaction,

(11) $$\begin{array}{c} K_{2}=\frac{n_{\text{total}\;}n_{\text{C}{\text{H}}_{4}}}{{\left(n_{{\text{H}}_{2}}\right)}^{2}}. \end{array}$$

Considering product gas as ideal gas, *K*_1_ and *K*_2_ can be expressed as a function of temperature [14], given by

(12) $$\begin{array}{c} K_{1}=\exp\left(\frac{5878}{T}+1.86\ln T-0.27\times 10^{-3}T-\frac{58200}{T^{2}}-18\right), \end{array}$$

(13) K2=exp⁡7082.842T−6.567ln⁡T+7.467×10−32T   s−2.167×10−66T2+0.0702×10−52T2+32.541.

Thus equilibrium composition of the product gas is obtained by simultaneously solving three linear equations (see (5)–(7)) and two nonlinear equations (see (9) and see (11)) in MATLAB platform using Newton-Raphson method.

Lower heating value of the dry product gas is estimated from the gas composition and is expressed in volume basis as [26]

(14) $$\begin{array}{c} \text{LHV}=10.79Y_{{\text{H}}_{2}}+12.26Y_{\text{CO}}+35.81Y_{{\text{CH}}_{4}}. \end{array}$$

Gasification efficiency of the process is given by

(15) ηgas =Energy  content  in  the  product  gasEnergy  content  in  biomass+Energy  content  in  steam.

The results of proximate and ultimate analyses of coconut shell are presented in Table 1.

## 3. Model Validation

**Figure 1:**
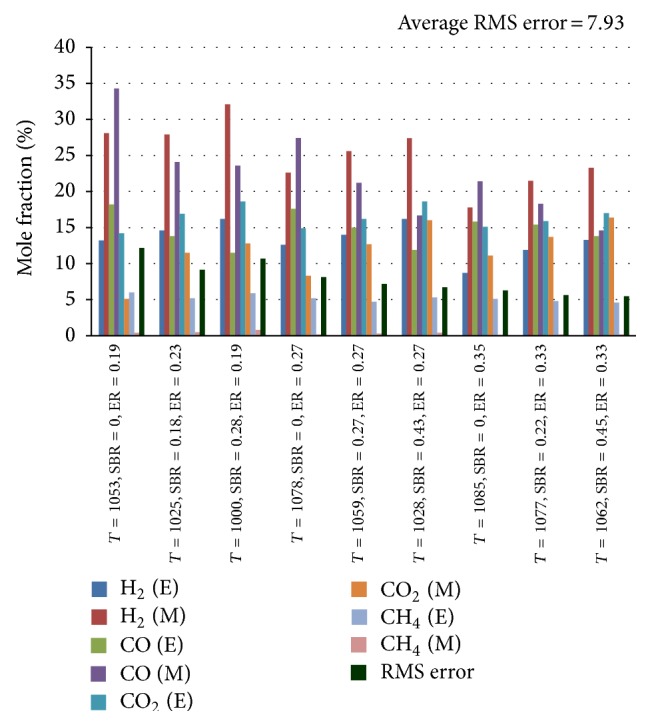
Comparison between experimental and model results. E: experimental result; M: model results.

Accuracy of the model is checked by comparing the predicted gas composition from the model with experimental results [27]. The error was estimated by using the statistical parameter of root mean square (RMS) error:

(16) $$\begin{array}{c} \text{RMS}=\sqrt{\frac{\sum {\left(X_{e}-X_{p}\right)}^{2}}{N}}, \end{array}$$

where *X*_*e*_, *X*_*p*_, and *N* are experimental data, predicted value, and number of observations, respectively. An average RMS value of 7.93 is obtained when the nine sets of experimental results are compared with their corresponding theoretical predictions, given in Figure 1.

## 4. Model Modification 

**Figure 2:**
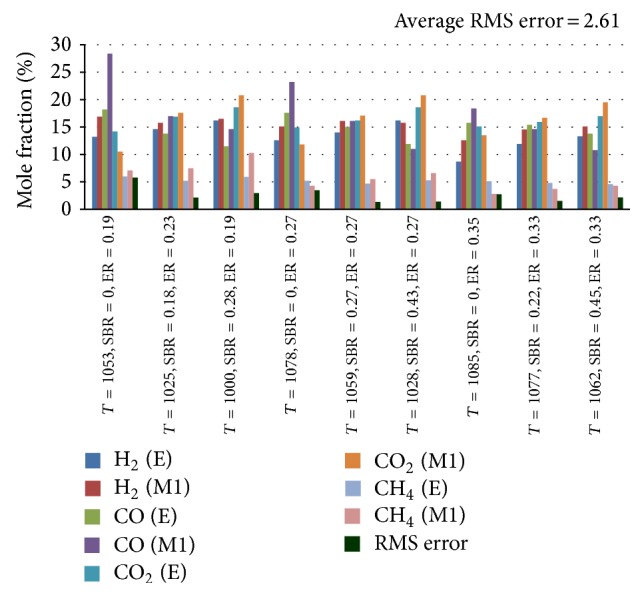
Comparison between experimental and modified model results. E: experimental result; M1: modified model results.

**Figure 3:**
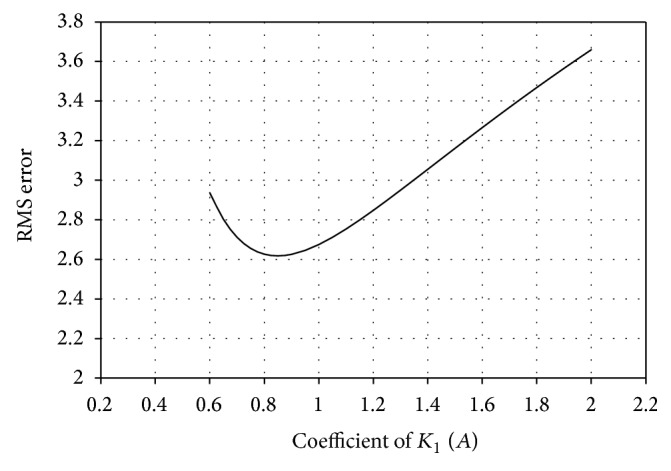
Variation of RMS error with coefficient of *K*_1_.

**Figure 4:**
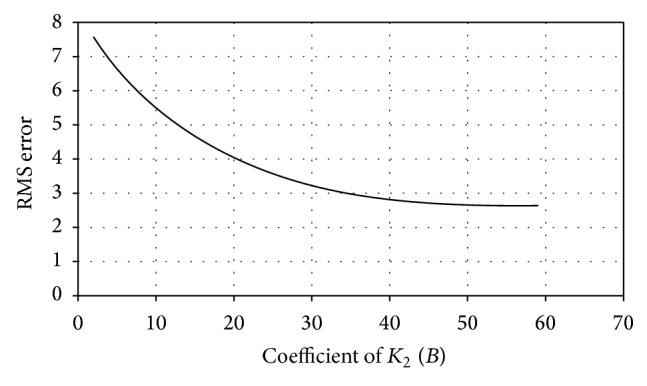
Variation of RMS error with coefficient of *K*_2_.

It is observed that H_2_ and CO concentrations were overpredicted and CO_2_ and CH_4_ concentrations were underpredicted by the present model from the experimental values. Similar results were observed by Melgar et al. [28] when they compared their model predicted gas composition with the experimental work of Jayah et al. [29]. Same results were obtained when the equilibrium models [30–33] were compared with the experimental results from fluidised bed steam gasification by Hofbauer et al. [34] and Rapagnà et al. [35]. The effect was also reported in [4, 22]. This deviation in concentration may be attributed to the existence of nonequilibrium conditions in the gasifier during the experiment. The model is upgraded to match the experimental results by multiplying with suitable coefficients *A* and *B* to *K*_1_ and *K*_2_, respectively [14]. The variation in RMS error is monitored by changing the values of *A* and *B* and the coefficient values corresponding to minimum RMS error are incorporated in the model for better prediction. The variation of RMS error with different values of *A* and *B* is shown in Figures 3 and 4, respectively. Incorporating suitable coefficients (*A* = 0.85 and *B* = 48), the average initial RMS error is reduced from 7.93 to a minimum of 2.61. A comparison between experimental results and that obtained from modified model is presented in Figure 2.

## 5. Model Application

The modified quasi-equilibrium model is used to predict the influence of key process parameters like temperature, steam to biomass ratio (SBR), and equivalence ratio (ER) on product gas composition, heating value, and gasification efficiency. Gasification study was conducted by keeping biomass mass flow rate as 1.0 kg/h and varying temperature and SBR and ER in the ranges of 800 to 1800 K, 0 to 2 and 0.15 to 0.4, respectively.

## 6. Results and Discussion

### 6.1. Effect of ER, SBR, and Temperature on Gas Composition

**Figure 5:**
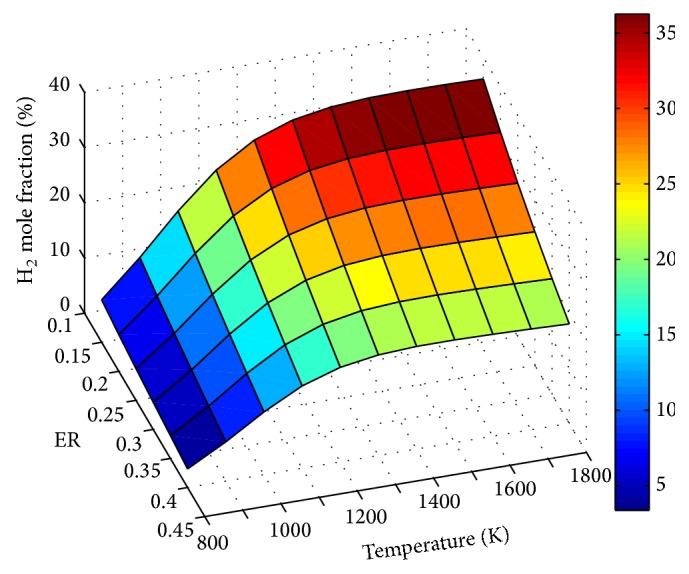
Effect of ER and temperature on H_2 _mole fraction (SBR = 1).

**Figure 6 fig6:**
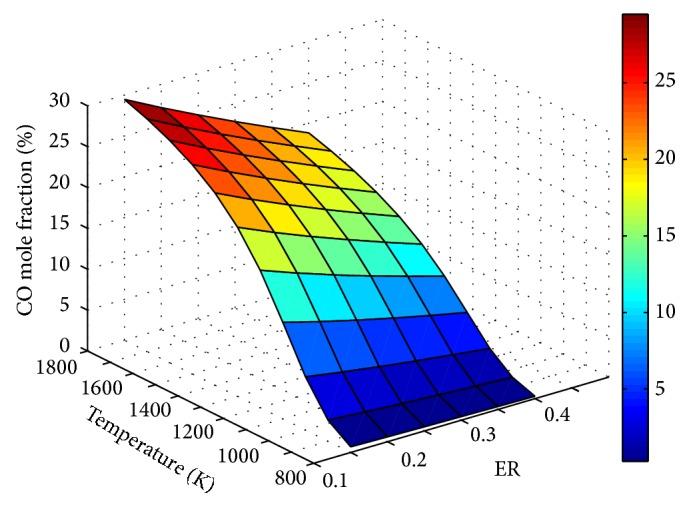
Effect of ER and temperature on CO mole fraction (SBR = 1).

**Figure 7 fig7:**
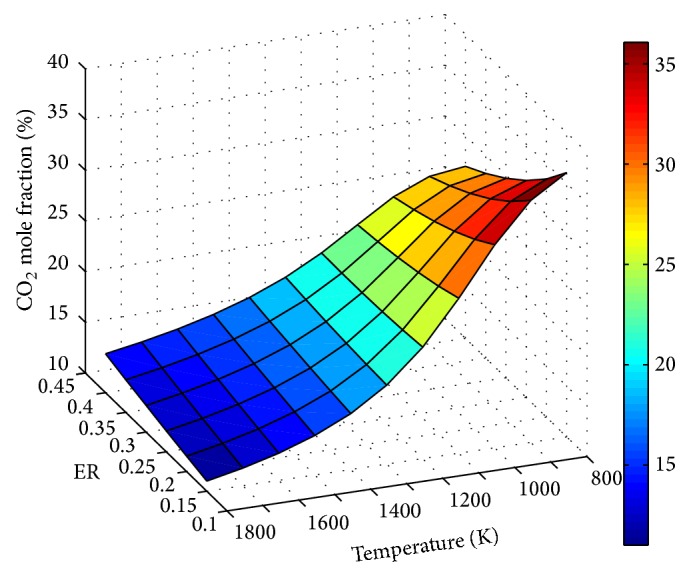
Effect of ER and temperature on CO_2 _mole fraction (SBR = 1).

**Figure 8:**
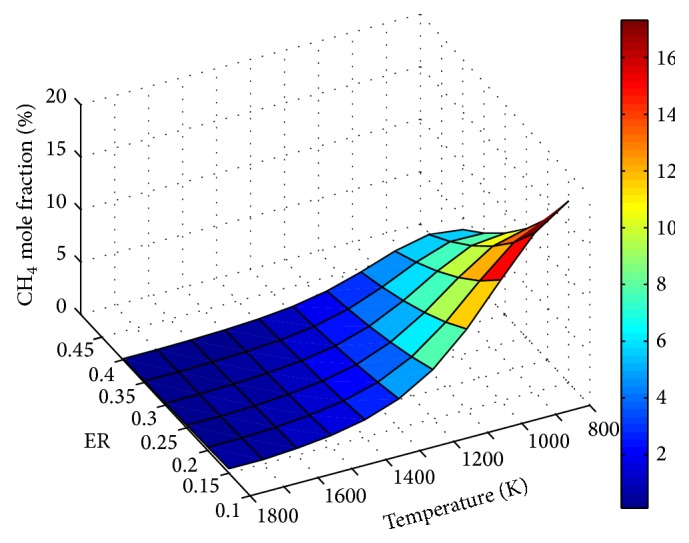
Effect of ER and temperature on CH_4_ mole fraction (SBR = 1).

The influence of ER and temperature on product gas composition is depicted through Figures 5 [Fig fig6] [Fig fig7]–8. It is observed that all the gas species except CO_2_ are decreasing with ER. This is due to shifting of the process more towards combustion by the addition of more and more air. Similar effect of ER on product gas composition was observed by Lim and Lee [20] and Puig-Arnavat et al. [22]. H_2_ mole fraction increases with temperature to a maximum value and then shows a slow and gradual decrease. The increase is more predominant at lower temperatures ranging from 800 to 1300 K, as shown in Figure 5. This is similar to the variation observed for H_2_ concentration by Lv et al. [36]

This trend of H_2_ is mainly due to the effect of exothermic water gas shift reaction. At higher temperature ranges reversal of the reaction, as per Le-Chatelier's principle, is responsible for the decrease in H_2_ mole fraction. The effect of shifting of endothermic reactions like methane reformation and water gas towards the product side is less pronounced to the reversal of water gas shift reaction. This may be the reason for the slight decrease of H_2_ concentration at higher temperature ranges. For SBR = 1, a maximum H_2_ mole fraction of 36.14% is obtained at a gasification temperature of 1500 K and ER of 0.15.

From Figure 6, it is clear that CO concentration increases with temperature and the rate of increase is more at lower temperature ranges. This is due to the combined effect of endothermic char gasification, water gas and methane reformation, and reversal of water gas shift reaction.

Effect of temperature on CO_2_ and CH_4_ concentrations is shown in Figures 7 and 8, respectively. Throughout, decrease of CO_2_ with temperature shows the dominance of endothermic Boudouard reaction on the process.

The decrease in CH_4_ with temperature is due to the combined effect of the shifting of endothermic steam methane reformation reaction and exothermic methanation reaction towards the product side and reactant side, respectively.

**Figure 9:**
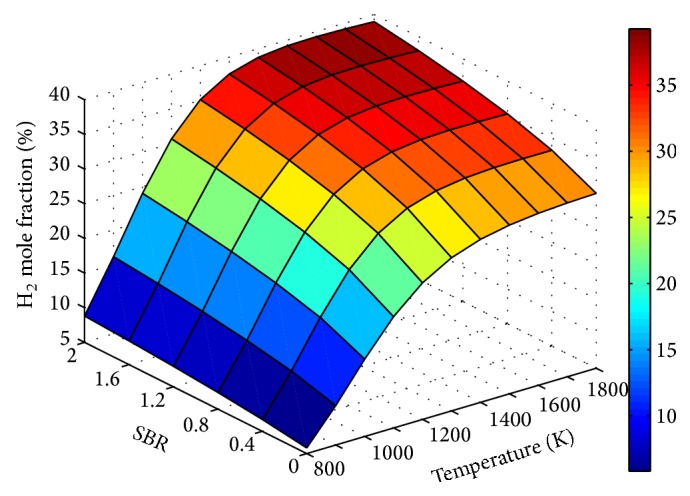
Effect of SBR and temperature on H_2_ mole fraction (ER = 0.15).

**Figure 10 fig10:**
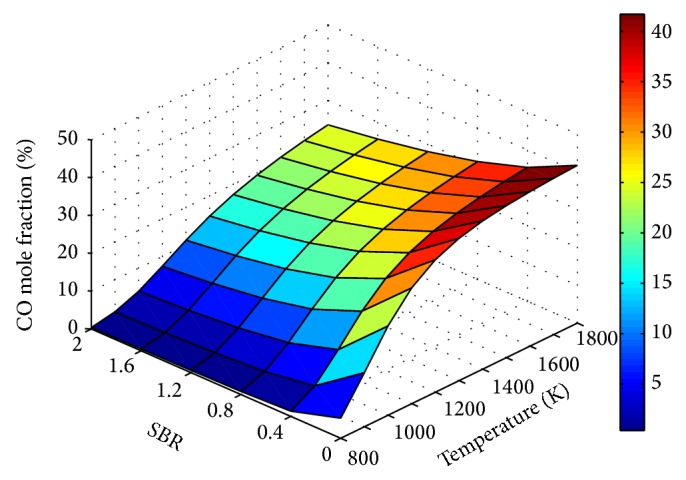
Effect of SBR and temperature on CO mole fraction (ER = 0.15).

**Figure 11 fig11:**
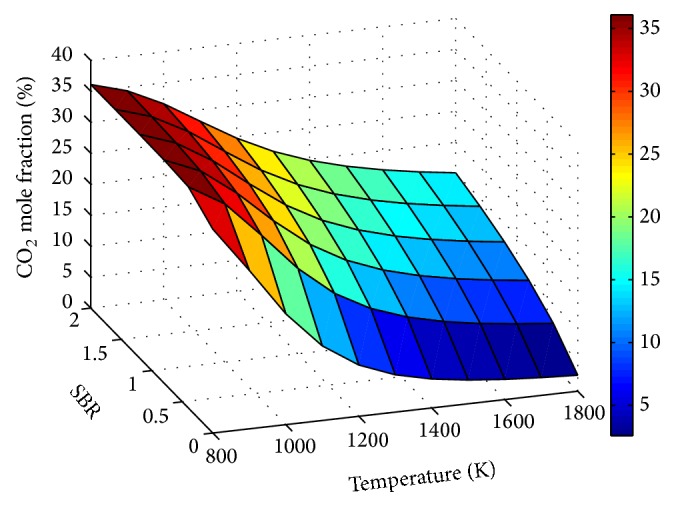
Effect of SBR and temperature on CO_2_ mole fraction (ER = 0.15).

**Figure 12:**
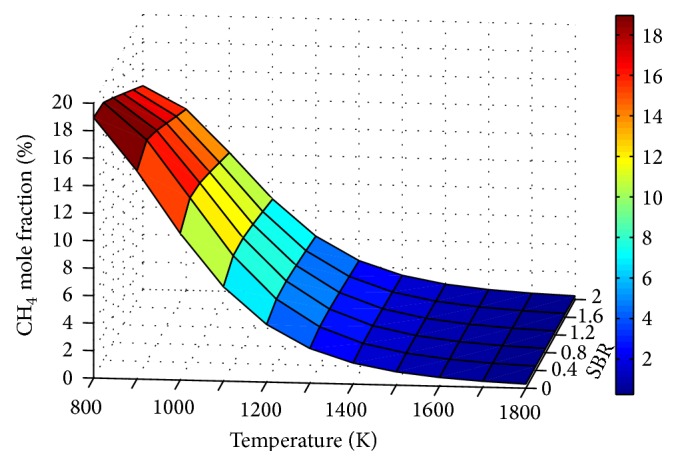
Effect of SBR and temperature on CH_4_ mole fraction (ER = 0.15).

Figures 9 [Fig fig10] [Fig fig11]–12 show the influence of SBR and temperature on product gas composition. H_2_ concentration seemed to increase throughout with SBR, but the rate of increase decreases gradually. This increase in H_2_ is due to the combined effect of water gas, steam methane reforming, and water gas shift reaction. When the SBR is increased from 0.8 to 1.2 the corresponding increase in H_2_ mole fraction is only 4%. Thus increasing SBR beyond a value of unity will not contribute much to hydrogen production compared to the energy spent for steam generation.

The adverse and favorable effect of steam addition on CO and CO_2_ mole fractions are depicted through Figures 10 and 11, respectively. The higher rate of CO_2_ increase at low temperature ranges is attributed to the effect of exothermic water gas shift reaction. Similar effect of increase in H_2_ and CO_2_ and decrease in CO molar concentrations with SBR at a temperature of 988 K and ER of 0.12 was observed in [37].

### 6.2. Effect of ER, SBR, and Temperature on Efficiency

**Figure 13:**
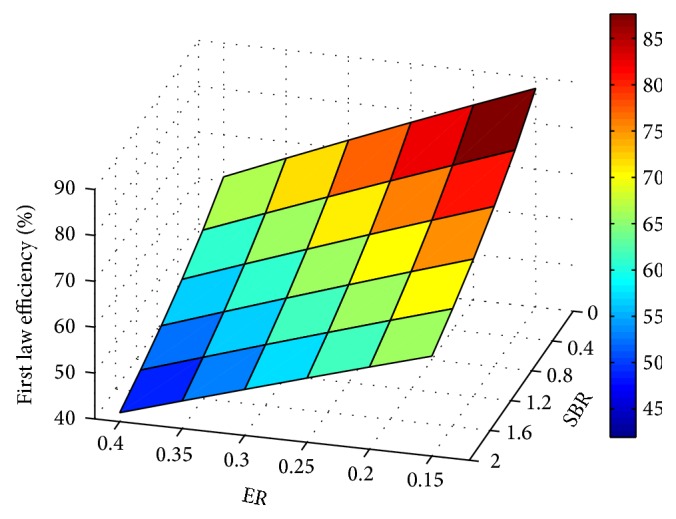
Effect of ER and SBR on efficiency (*T* = 1500 K).

**Figure 14:**
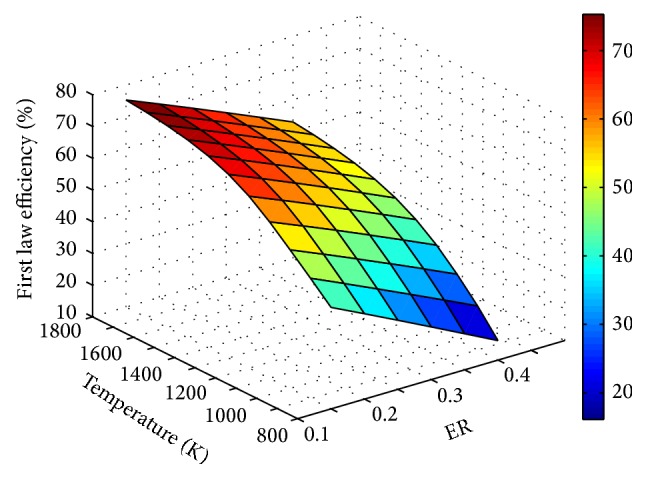
Effect of ER and temperature on efficiency (SBR = 1).

**Figure 15:**
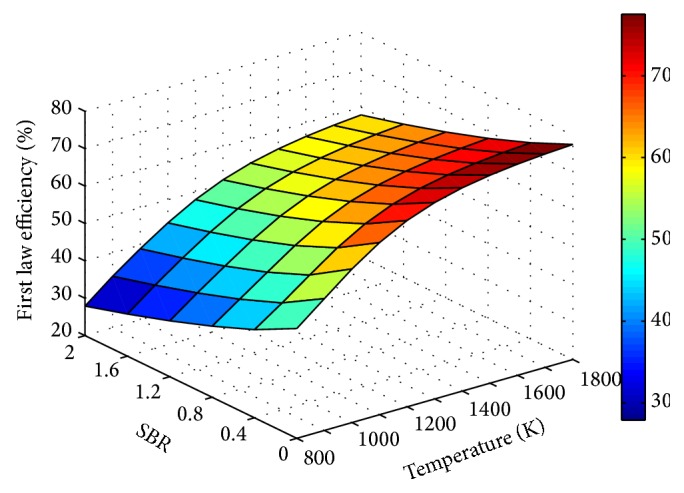
Effect of temperature and SBR on efficiency (ER = 0.15).

Influence of process parameters on gasification efficiency is shown through Figures 13, 14, and 15. For any fixed values of ER and temperature, efficiency is observed to decrease with SBR.

The decrease in efficiency with increase in SBR is due to the increased energy input in the form of steam, whereas the reason for efficiency degradation with ER is the reduced LHV of the product gas. Product gas composition, LHV, and gas yield predicted using the modified model at different operating conditions are given in Table 2. It is observed that irrespective of ER and SBR values, H_2_ concentration is more for 1500 K.

## 7. Conclusion

A thermodynamic equilibrium model was developed to analyse air steam gasification of biomass. The developed model was compared with experimental results for product gas composition and its prediction accuracy is improved by multiplying equilibrium constants with suitable coefficients. The modified quasi-equilibrium model is used to conduct parametric study and first law analysis on air steam gasification of coconut shell. For an SBR of unity, the maximum mole fraction of hydrogen in the product gas was found to be 36.14% with a lower heating value of 7.49 MJ/Nm^3^ at a gasification temperature of 1500 K and ER of 0.15.

## Figures and Tables

**Table 1:** Proximate and ultimate analyses of coconut shell.

Proximate analysis (wt.%)		Ultimate analysis (wt.%)	
Moisture	8	C	45.61
Volatile matter	71	H	5.61
Ash	4	O	48.16
Fixed carbon	17	N	0.26
		S	0.34

**Table 2:** Product gas composition, LHV, and gas yield at different operating conditions.

*T* (K)	ER	SBR	Product gas composition (% dry basis)					LHV (MJ/Nm^3^)	GAS YIELD (Nm^3^/h)
			H_2_	CO	CO_2_	CH_4_	N_2_		
800	0.2	0.4	5.93	1.15	33.25	14.8	44.47	6.08	1.47
		1.6	7.16	0.41	33.48	12.09	45.26	5.15	1.41
	0.3	0.4	4.58	0.82	29.59	8.92	55.69	3.79	1.76
		1.6	5.08	0.29	29.58	6.59	56.86	2.94	1.69
	0.4	0.4	3.35	0.55	26.98	4.78	63.94	2.14	2.05
		1.6	3.2	0.18	26.83	2.77	65.42	1.35	1.96

1300	0.2	0.4	27.44	25.32	13.28	2.18	31.38	6.93	2.07
		1.6	32.54	15.00	20.48	2.08	28.3	6.14	2.21
	0.3	0.4	21.22	20.44	14.48	1.29	42.17	5.33	2.32
		1.6	25.52	12.17	20.37	1.30	39.04	4.75	2.43
	0.4	0.4	15.97	16.13	15.74	0.72	51.04	4.01	2.56
		1.6	19.48	9.61	20.44	0.77	48.1	3.59	2.65

1500	0.2	0.4	28.87	29.53	10.00	0.78	30.82	7.12	2.14
		1.6	33.91	19.57	16.95	0.746	28.82	7.15	2.28
	0.3	0.4	21.86	24.36	11.57	0.44	41.77	5.59	2.36
		1.6	26.45	16.16	17.41	0.46	39.52	5.70	2.50
	0.4	0.4	16.15	19.67	13.18	0.24	50.76	4.31	2.59
		1.6	20.15	13.04	17.96	0.27	30.82	3.91	2.71

1800	0.2	0.4	28.85	32.62	7.70	0.23	30.18	7.31	2.15
		1.6	33.61	23.51	14.05	0.21	27.02	6.66	2.30
	0.3	0.4	21.46	27.44	9.34	0.12	41.24	5.82	2.37
		1.6	25.99	19.73	14.82	0.13	37.73	5.34	2.51
	0.4	0.4	15.53	22.59	11.07	0.06	50.35	4.55	2.60
		1.6	19.66	16.23	15.68	0.07	46.76	4.19	2.72
